# Quality by Design as a Tool in the Optimisation of Nanoparticle Preparation—A Case Study of PLGA Nanoparticles

**DOI:** 10.3390/pharmaceutics15020617

**Published:** 2023-02-12

**Authors:** Anna-Maria Struzek, Regina Scherließ

**Affiliations:** 1 Department of Pharmaceutics and Biopharmaceutics, Kiel University, 24118 Kiel, Germany; 2 Priority Research Area Kiel Nano, Surface and Interface Sciences (KiNSIS), Kiel University, 24118 Kiel, Germany

**Keywords:** quality by design, design of experiment, screening design, central composite response surface design, quality attributes, double-emulsion solvent evaporation method, protein drug delivery

## Abstract

Nanoparticles can be used as drug carriers in various applications (e.g., in pulmonary drug delivery and mucosal vaccination). For further investigations, such as drug release studies, as well as for cell and tissue targeting, particles with defined properties are needed. The purpose of the study was to show a multi-step systematic method utilising quality by design to ensure the quality of ovalbumin loaded polylactic-co-glycolic acid nanoparticles (OVA-PLGA-NP), which can be delivered to the lung, and to gain knowledge of the preparation method (double-emulsion solvent evaporation method) in an early development process. Within a definitive screening design, several process parameters (OVA, PLGA and stabiliser concentrations, stirring time and stirring speed of inner emulsion and stirring time and stirring speed of double emulsion) were varied to analyse their impact on resulting properties (z-average, PDI, loading efficiency and loading capacity). The results showed that the preparation of the inner emulsion mainly influenced the drug loading, while the parameters of the second emulsifying step controlled the size. Then a central composite response surface design was used to achieve a predictable OVA-PLGA-NP with an average particle size of 700 nm and high drug-loading. This also enabled the demonstration of curvature and interaction of the stabiliser and the PLGA concentration.

## 1. Introduction

Since many pathogens enter the body via the lung, its mucosa is equipped with numerous immunocompetent cells [1]. They form the pulmonary mucosal immune system, which protects the human body mainly against respiratory pathogens, such as SARS-CoV-2. The activation of the immune system can also been used for therapeutic vaccination, which is very efficient for mucosal immune response [2]. It has been discovered that particulate antigens are more immunogenic than purified proteins [3]. Furthermore, mucosal vaccines are most effective when they mimic pathogens in certain key characteristics, such as particle size and surface characteristics [2]. For antigen protection and enhanced uptake, the antigen can be encapsulated in small microparticles or nanoparticles (NP) [3]. Physical protection and particulate delivery of the antigen is mandatory. Polymers are the most stable materials for microcarriers and nanocarriers [4]. Generally, the uptake of polymeric nanoparticles (PNP) is not only controlled by the route of application but also by the particle size and shape as well as charge and functional groups on the surface [5]. Depending on use and target cell, controlled modification of particle properties is necessary to deliver the drug to the target location. To produce these tailored particles, it is essential to understand the preparation process and its influence on the final product. This enables the guidance and control of the production.

Despite many advances in laboratory-scale PNP preparation, there are still many challenges in the manufacturing process, such as control and replication of key characteristics of the desired nanoparticles [6], that make it difficult to produce high-quality PNP. Preparation processes for PNP are oftentimes multi-step bulk processes with many influencing variables rendering the preparation itself comparatively complex. In order to ensure the ultimate quality of the product early in the development of the method and to understand as early as possible which parameters influence the process, the use of quality by design (QbD) is essential, and requested by ICH.

Quality by design is defined as systematic approach within pharmaceutical development [7]. It includes the evaluation and understanding of the formulation and manufacturing process. The goal is to develop a controlled process that ensures the quality of the product [7,8,9,10]. The ICH guideline Q8(R2) describes different approaches and tools to implement QbD in practice such as “multivariate experiments”, “statistical process control methods” also known as design of experiment and a “risk-based control strategy” [7].

In the present study, polylactic-co-glycolic acid nanoparticles (PLGA-NP) loaded with ovalbumin (OVA) serve as examples to show the application of QbD in process development. Polylactic-co-glycolic acid (PLGA) is one of the most used polymers in the research of nanoparticulate drug delivery systems [11]. It is a water insoluble, biodegradable and biocompatible polymer which has been proven to be a safe, non-active ingredient by U.S. FDA [12,13]. PLGA nanoparticles can be used for drug delivery of hydrophilic as well as hydrophobic actives. While for hydrophobic actives a one-step precipitation method or a single emulsion method can be used, for hydrophilic actives a double-emulsion preparation method is more suitable [12]. 

Such a double-emulsion solvent evaporation method for the preparation of OVA-loaded PLGA-NP was selected for QbD analysis and optimisation within the studies presented in this paper. For a guided optimisation, it is important to find those process parameters whose influence on the final product is large enough to change its properties in a targeted manner. For the systematic investigation of the process, the procedure was divided into the following steps:

1. Definition of quality attributes and selection of outcome parameters;
2. Identification and evaluation of process variables that might have an impact on PLGA-NP properties;
3. Identification of significant process parameters by screening of the most promising variables;
4. First approach of optimisation using the results of the screening;
5. Further optimisation and controlled adjustments of the products quality by varying the most influencing process parameters within a response surface design;
6. Prediction and verification of the optimal process parameters.

The first and second step are parts of the planning phase, the third and fourth step are parts of the screening phase, the fifth step is the optimisation phase, and the last step is the verification phase.

## 2. Materials and Methods

### 2.1. Nanoparticle Preparation

PLGA-Nanoparticles were prepared by a double emulsion solvent evaporation method [14] using an ultra turrax^®^ (T25, IKA Labortechnik, Staufen, Germany) for emulsification steps. For the inner emulsion, Ovalbumin (albumin from chicken egg white, lyophilised powder ≥98%, Sigma-Aldrich, St. Louis, MO, USA) was dissolved in phosphate-buffered saline pH 7.4 (European Pharmacopoeia. 10.0/4.1.3; 4005000), obtaining the internal aqueous phase (W1). PLGA (Resomer^®^ RG 503 H, Evonik, Darmstadt, Germany) was dissolved in ethyl acetate (HPLC grade, Carl Roth, Karlsruhe, Germany) for the organic phase (O). W1 and O were combined and emulsified in a 15-mL-centrifuge tube before they were added to the external aqueous phase (W2), consisting of polyvinyl alcohol (PVA) (Mowiol 4-88, Sigma-Aldrich, St. Louis, MO, USA) in ultrapure water (Direct-Q^®^, Merck, Darmstadt, Germany), in a 50-mL-centrifuge tube. Then, the inner emulsion and W2 were emulsified resulting in a W/O/W double emulsion. The double emulsion was added to the stabiliser solution and stirred at 400 rpm on a magnetic stirrer (IKA^®^ Ro 15, IKA Labortechnik, Staufen, Germany). Ethyl acetate was evaporated under continued stirring at 400 rpm overnight. The product was a nanoparticle suspension. Table 1 lists all compositions and quantities, all of which were held constant.

### 2.2. Purification

To eliminate non-encapsulated OVA and excess stabiliser, the OVA-PLGA-NP were purified as follows: To avoid non-reversible agglomeration, three centrifugal steps were used to separate the NP from the supernatant. The nanosuspension was centrifuged for 10 min at 3500 rpm (1438 rcf) (Centrifuge 5430 R, Eppendorf AG, Hamburg, Germany). The decanted supernatant was centrifuged for 10 min at 5500 rpm (3551 rcf). Then the decanted supernatant of the second step was centrifuged for 30 min at 7830 rpm (7197 rcf), resulting in a clear supernatant. The three pellets containing the NP were pooled, resuspended in 2 mL water, and filled up to 50 mL. The three centrifugation steps were repeated and the resuspended pellets were again pooled and filled up to 15 mL with water. All centrifugation steps were performed at 4 °C.

### 2.3. Measurement of Size, Size Distribution and Zeta-Potential

Z-average, polydispersity index (PDI) and zeta potential were determined by dynamic light scattering (DLS) with the Zetasizer ZS Nano (Malvern Instruments, Malvern, UK) at 25 °C and an equilibration time of 120 s. The samples were not diluted. All results are the mean of three measurements. The viscosity of every nanosuspension was measured before and included in the calculation (SV-10 Vibro-viscometer, Malvern Instruments, Malvern, UK).

### 2.4. Protein Quantification 

To analyse the ovalbumin content in the nanoparticles, the Micro BCA Protein Assay Kit and the BCA Protein Assay Kit (both Thermo Fisher Scientific, Waltham, MA, USA) were utilised. The same ovalbumin batch was used for calibration and samples. PLGA-NP were dissolved in 0.2 M NaOH at 37 °C for 2 h. Then, the samples were neutralised with 0.2 M HCl. The (Micro) BCA Assay was performed as described in the assay instruction. For the BCA Assay 25 µL of each standard and sample were pipetted to a 96-well microplate well. A quantum of 200 µL working reagent (50:1, Reagent A:B) was added, and the two solutions were mixed by shaking. The covered plate was incubated at 37 °C for 30 min. For the Micro BCA Assay 150 µL of each standard and sample were pipetted to a 96-well microplate well. A quantum of 150 µL working reagent (25:24:1, Reagent MA:MB:MC) was added, and the two solutions were mixed by shaking. The covered plate was incubated at 37 °C for 2 h. For both assays the absorbance was measured at 562 nm against a reference blank. The encapsulated amount of OVA was determined via a calibration curve. This is in turn was needed to calculate the loading efficiency (LE) (1) and the loading capacity (LC) (2).

Loading efficiency

(1) $$\mathrm{Loading}\;\mathrm{efficiency}\;\left(\%\right)=\frac{m\left(\mathrm{encapsulated}\;\mathrm{OVA}\right)}{m\left(\mathrm{total}\;\mathrm{OVA}\right)}\times 100\;\%$$

Loading capacity

(2) $$\mathrm{loading}\;\mathrm{capacity}\;\left(\%\right)=\frac{m\left(\mathrm{encapsulated}\;\mathrm{OVA}\right)}{m\left(\mathrm{encapsulated}\;\mathrm{OVA}\right)+m\left(\mathrm{total}\;\mathrm{PLGA}\right)}\times 100\;\%$$

### 2.5. Scanning Electron Microscopy (SEM)

For SEM, air-dried samples were sputtered with gold, utilising a Bal-Tech SCP 005 Sputter Coater (Bal-Tec AG, Balzers, Lichtenstein) for 40 s. SEM pictures were taken with a Phenom Pro XL (Thermo Fisher Scientific Inc., Waltham, MA, USA) at a working voltage of 10 kV.

### 2.6. Design of Experiements

To generate the design of experiments (DoE) setup, Minitab statistic software version 18 (Minitab Inc., State College, PA, USA) was used. The experiments for all DoE were conducted in randomised order with one replicate. Parameters, which were not changed, should be as constant as possible. In this study, a screening design and a follow up response surface design were used to optimise the nanoparticles and to understand the relation between parameter settings and outcomes. 

Screening designs are intended for the early stage of an investigation when many factors are still included in the study [15]. Those designs help to identify the main effects as well as the factors that have the largest overall effects and factors which have no significant effect within the experimental design space.

A definitive screening design (DSD), which we used in this study, has three factor levels [15]. Those screening designs have several benefits: Most important second-order effects do not bias the estimation of main effects. In addition two-factor interactions or quadratic effects can be estimated if the true effects are much larger than the error standard deviation [16]. 

In this study, the process variables we identified in the previous step were examined in a DSD. The main effects should be detected and not statistically significant process parameters should be eliminated in further experiments. The most promising variables should be used as parameters for the response surface design. 

The response surface methodology (RSM) can create a more dimensional map (response surface) correlating the parameters and the outcome and include interactions of parameters as well as curvature [17]. Even with a comparatively small number of runs, RSM can provide a good predictability for future outcomes within the experimental region [18] and determine the optimum settings of parameters that result in the optimal response [19]. 

A central composite design (CCD) is a complete fractional design expanded by several centre points and axial points, which are positioned at the axis of each parameter at the distance of alpha from the centre point [19]. Since every parameter is probed at five levels, a CCD results in a good resolution for the response surface. In this study, the design was utilised for further optimisation of NP to improve size adjustment, to maximise LE and to narrow particle size distribution.

## 3. Results and Discussion

When starting a QbD experimental setup, a target product profile needs to be specified. Depending on the application of the NP drug carrier systems, specific requirements are defined, which are important for selection of variables and later optimisation of process parameters.

### 3.1. Definition of Quality Attributes and Selection of Outcome Parameters

To achieve a pharmaceutical effect, it is important that the nanoparticulate carrier brings sufficient API to or into the target cell and releases it there. As previously discussed, the particle size is the decisive quality attribute when it comes to distribution in the body, reaching of target cells, and cellular uptake. As outcome parameters for the DoE, the z-average (average particle size) was chosen for its importance to reach the target cell, the PDI as indicator for size distribution, the LC for later potency of the product and the LE for process cost efficiency. It was defined for the purposes of this study that desired NP should have a z-average of 700 nm to only allow uptake in phagocytosing cells and a PDI below 0.3, because this indicates a monodisperse size distribution [20]. The LE and LC were to be as high as possible.

### 3.2. Identification and Evaluation of Process Parameters

To choose parameters for DoE a comprehensive identification of all variables of the preparation process should be performed [7]. To minimise the lack of variables a risk assessment tool can be used. After creating an overview of variables as completely as possible, a thoughtful evaluation of the variables should take place. The potential influence of a variable on the outcomes should dominate the evaluation. Additionally, the practicability, effort and detectability should be taken into consideration to exclude purely theoretical results.

**Figure 1:**
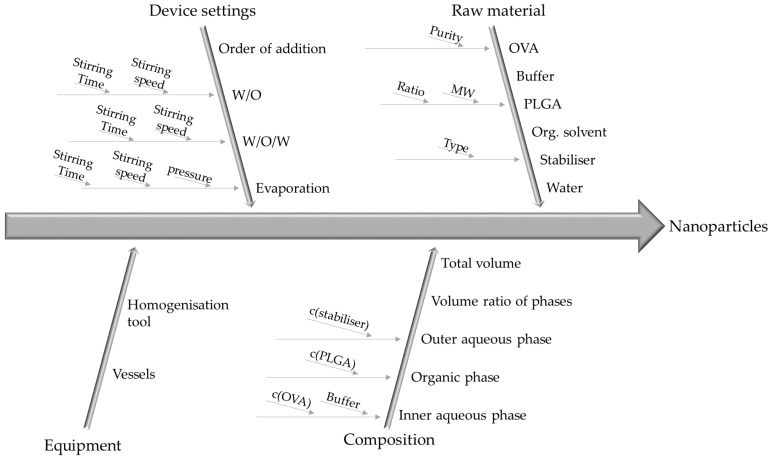
Fishbone (Cause-and-effect) diagram of process parameters, which potentially have an influence on the defined outcomes.

To identify all variables, which might influence the product quality, a fishbone cause and effect diagram was used (Figure 1). As major causes the raw materials, the composition of the formulation, the device settings and the equipment were identified. For each main cause, several sub-causes were determined (Figure 1).

In the case of a multi-step preparation with many variables, it is hardly possible to examine all variables as parameters in an experimental design with a reasonable amount of effort. The next step was to evaluate all variables and eliminate those whose follow-up would be less promising, as scientific rationale expects them to be of negligible influence. The variables that have not been investigated during the experimental design remain with the status “criticality unknown”.

Due to laboratory equipment, the homogenisation tool and vessels were not changed. For this study, we decided to fix the type and quality of raw materials as parameters for two reasons. The different raw materials were already investigated in former studies and the types of raw materials were already well chosen [14]. Resomer^®^ RG 503 H has a glass transition temperature of 44–48 °C and therefore is stable at body temperature. The ratio of lactic acid and glycolic acid favours a good drug release without loss of stability. For the double-emulsion-solvent-evaporation method, it is essential that the organic solvent is safe, volatile and not miscible with water, which limits the choice of the organic solvent. The second reason is that changing the quality of raw materials may have a relatively small influence on the defined outcome parameters. The only raw material that might have an influence on the size is the type of stabiliser, but the stabiliser concentration may have the bigger influence. Thus, concentration was assessed for a fixed type of stabiliser, namely PVA. PVA is a safe stabiliser and is solid at room temperature, what makes further processing easier.

Former experiments of single- and double-emulsion solvent evaporation methods for different nanoparticles showed that process variables as homogenisation time and intensity (here, rotation speed) may have an influence on size and drug loading [21,22]. Therefore, the stirring speed and stirring time for inner emulsion as well as double emulsion were selected as parameters. 

Even though the evaporation rate would be a promising parameter [13], it was not included in the DoE, to keep the equipment setup simple and the experimental design fast. One way to evaporate the organic solvent is by using a rotary evaporator, where the pressure and rotation speed can be varied. In this case every sample must have been treated separately, which leads to a high effort. The other way is to stir the samples at normal environmental conditions for a longer time. Due to the much lower effort and more simple equipment, this technique was chosen.

It was also shown earlier that the composition of the double emulsion has a major influence; Mainardes et al. investigated the concentration of stabiliser, the polymer content and the volume ratio of the phases, concluding that those composition parameters effect the formation of PLGA nanoparticles. Additionally, the API concentration may have an influence on drug loading and loading efficiency [22]. Since it is not possible to change the concentration, the mass and the volume independently, we had to decide which should be changed and which should be held constant. Since the volume is limited by the vessel and the minimal depth of immersion, it was set as constant. Thus, the concentration is equivalent to the mass (3).

(3) $$c=\frac{m}{V}$$

Because of these reasons, the following variables were chosen as parameters for further investigations: OVA concentration, PLGA concentration, stabiliser concentration, stirring time and speed of preparation of the inner emulsion and stirring time and speed of preparation of the double emulsion. 

### 3.3. Identification of Significant Process Parameters by Screening Design

#### 3.3.1. Definitive Screening Design

Since seven parameters were chosen for further investigation, a screening design was reasonable. The Ultra Turrax^®^, which we used for homogenisation, had five settings for the stirring speed; thus, it was treated as a categorical parameter. The Minitab software generated the DSD with five numerical variables which have three levels each, and two categorical variables with only two levels (Table 2), resulting in 18 runs with two center points.

**Figure 2:**
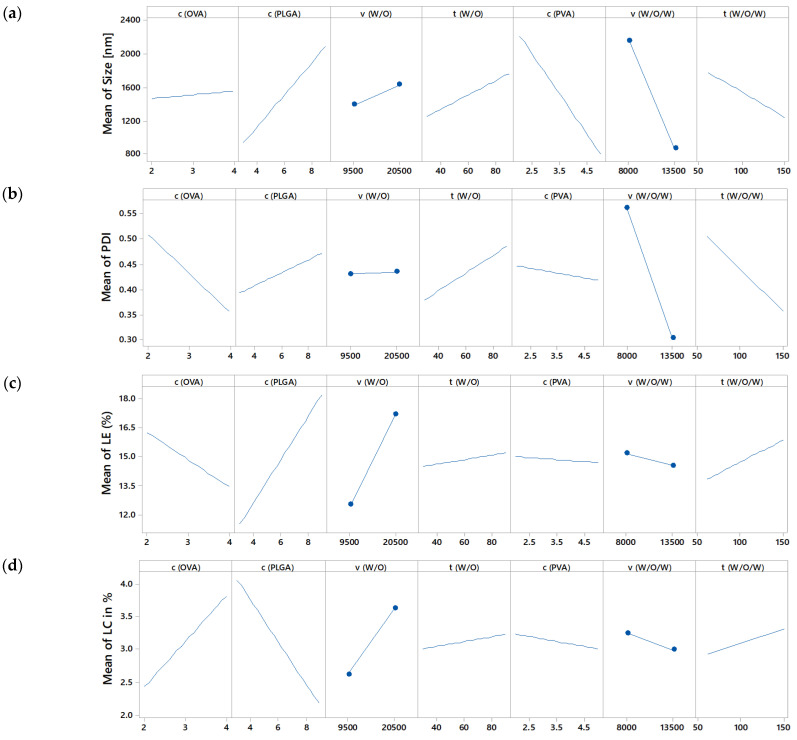
Main effects plots for z-average (**a**), PDI (**b**), loading efficiency (**c**) and drug load (**d**).

The experiments according to the definitive screening design were carried out in randomised order as generated by Minitab software, and the responses were determined (Supplementary Information). The design was analysed for every outcome using linear terms and a two-sided confidence level for all intervals of 95%. The *p*-values for every outcome model were <0.05, leading to the conclusion that the models explain variation in the response [23]. The residual plots were inconspicuous, and in the residual versus variable plots no pattern could be detected. This indicates that no square terms or interactions are missing. Therefore, the models were used for process analysis without further modification. The significances of all parameters on the outcome were compiled in Table 3. From those results, all main effect plots are generated to visualise the effect of the parameters on their response (Figure 2). 

#### 3.3.2. Effect on Size and Size Distribution

The z-average varied between 374.53 nm (run 15) and 5565.00 nm (run 4). The z-average was mainly affected by the significant parameters, namely the stirring speed of the double emulsion, stabiliser concentration and PLGA concentration (Table 3). While a higher stirring speed and higher stabiliser concentration led to smaller droplets and therefore to smaller particles (Figure 2a), the PLGA concentration defined the nanoparticle size after evaporation. A higher PLGA concentration led to larger particles (Figure 2a). Preparation parameters of the inner emulsion had no significant influence on particle size, size distribution and the OVA concentration (Table 3). Unexpectedly, stirring time does not have a significant influence on the z-average (Table 3). Such results need to be evaluated carefully, as it might be possible that with higher variation of this parameter the effect might become significant. It is also possible that the particle size reaches a plateau and even a longer stirring time would lead to no further effect. This experiment showed that controlling the PDI was difficult. Due to the similarity of the effect plots of z-average and PDI (Figure 2a,b) it can be assumed that adjustments, which were leading to smaller particles, also led to a smaller PDI and vice versa. It might not be possible to create large particles with a small PDI within this setup.

#### 3.3.3. Effect on Drug Loading and Loading Efficiency

Within the DoE the LE varied in a range from 7.22% to 23.95% and the drug loading varied in a range from 1.32% to 6.64%. While the stirring speed of the inner emulsion and the PLGA concentration affected LE and LC in the same direction (positive slope), the OVA concentration had a reverse effect for both outcomes (Figure 2c,d). A higher OVA concentration led to lower LE but higher LC, meaning that with more OVA used, more OVA was entrapped in NP in absolute terms, but less in relation to the amount used. A higher stirring speed of the double emulsion led to a higher redistribution of OVA from the internal aqueous phase to the external aqueous phase resulting in a lower LE and lower LC (Figure 2c,d).

### 3.4. First Approach of Optimisation with Screening Results 

Although a screening is not intended for accurate adjustment, it is possible to optimise the response and to use it as starting point for further experiments. Before creating a follow up DoE, the product can be prepared with “optimised” process parameters to confirm the reliability of the screening DoE. 

Using the response optimiser of Minitab 18, the following parameters were suggested for a target size of 700 nm, minimised PDI and maximised drug loading: c(OVA) = 3.9%; c(PLGA) = 9%; v(W/O) = 20,500 rpm; t(W/O) = 68 s; c(PVA) = 5%; v(W/O/W) = 13,500 rpm; and t(W/O/W) = 150 s. The prognosis for the outcomes, which was calculated from the model equations (not shown), can be seen in Table 4 as well as in the results of the NP, which have been produced with those settings. The outcome of the preliminary optimisation was already fitting well (Table 4). This approach increases confidence in the model.

### 3.5. Further Optimisation by Response Surface Design

Since a screening design is not intended for accurate prediction, a higher-resolution design should be used as the next step to increase insight into influences and create a good predictability.

#### 3.5.1. Central Composite Response Surface Design

A CCD was chosen for its ability to optimise a response and to create a response surface with curvature (non-linear correlations). Since the number of trials (N) increases exponentially with the number of factors (k) and linearly with the number of centre points (N_0_) (4), it is recommended to limit the number of tests when the number of factors is increasing [24] or to choose less variables as parameters, in order not to blow up the number of experiments.

N = 2^k^ + 2k + N_0_ (4)

For the PLGA-NP being the model setup, size control was a major goal, so the PLGA and PVA concentrations were chosen as parameters due to their prominent influence on this parameter. Since the stirring speed could not be adjusted continuously, it was unsuitable for precise size adjustment, despite its great influence, and was therefore kept constant.

All samples were prepared with an OVA concentration of 3.94% at 20,500 rpm, 68 s for inner emulsion and 13,500 rpm and 150 s for outer emulsion, as derived from the previous DoE. The PLGA and PVA concentrations were varied from 6.17 to 11.83% and from 2.17 to 7.83%, respectively (Table 5), resulting in thirteen total formulation runs with four cube points, four axial points and five centre points. Alpha was 1.414 leading to a distance from the centre point of 2.83% for PLGA and PVA concentration. 

**Figure 3:**
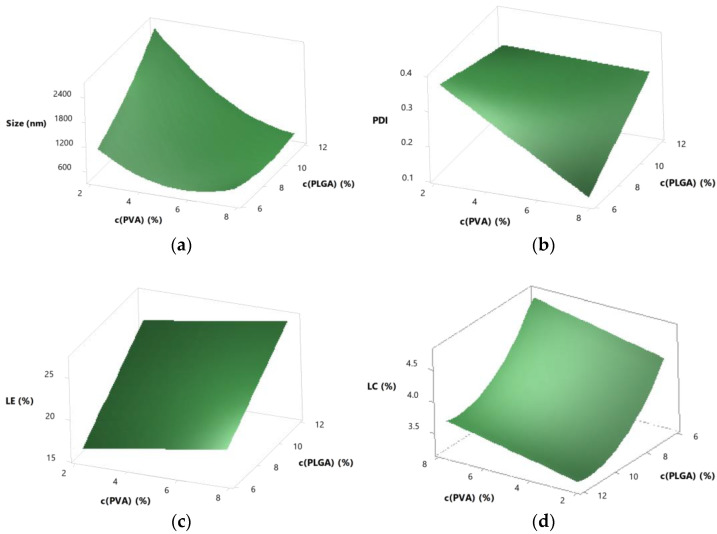
Response surface plots of z-average (**a**), PDI (**b**), LE (**c**) and LC (**d**), in dependence on PVA concentration in % and PLGA concentration in %.

Table 6 shows all results of the CDD. Regression equations were calculated using backward elimination and response surface plots were generated (Figure 3) for all outcomes using the Minitab Software.

#### 3.5.2. Interactions

**Figure 4:**
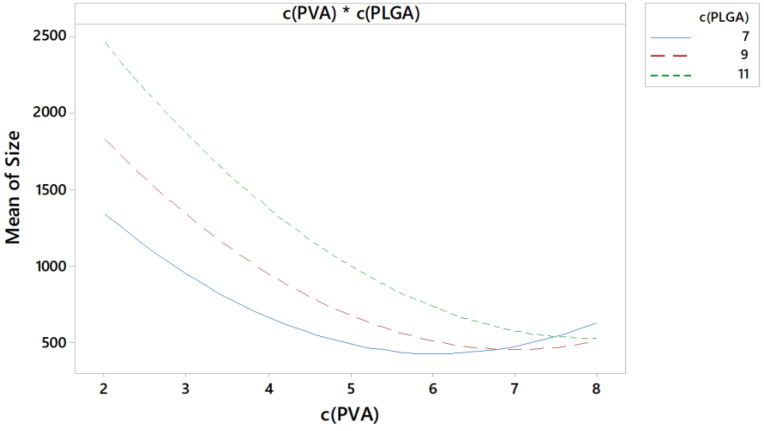
Interaction plot for z-average as a function of stabiliser concentration in % for 7% (blue), 9% (red) and 11% (green) PLGA.

In addition to the linear terms, the quadratic and two-way interaction terms were also significant (*p* < 0.05) for the size. That means that the response surface contained curvature in both directions and indicates an interaction of the factors [25,26]. Both curvature and divergent slope can be seen in Figure 4. All three lines flatten at a higher stabiliser concentration. That could indicate e.g., a saturation with stabiliser for different PLGA concentrations. The lower the PLGA concentrations, the less stabiliser is needed for saturation of the interface between inner and outer phase. 

#### 3.5.3. Predictability 

Predicted R² (R² (pred)) is a value that indicates how well future values can be predicted from the model. The larger an R² (pred) is, the better is the predictability of the model [27]. For the CCD the following R² (pred) were determined: 94.50% for z-average, 38.61% for PDI, 68.64% for LE and 67.33% for LC. While the size could be predicted very well and was therefore easily adjustable, the PDI showed a poor predictability. Even this optimised process was not suitable for changing the PDI in a targeted way. The R² (pred) for LE and LC was sufficiently good to provide predictability.

#### 3.5.4. Optimisation

The preparation method was optimised to achieve particles of a size of 700 nm, a minimal PDI and a maximal LE. This resulted in a PLGA concentration of 11.7% and a PVA concentration of 6.7% to be used in the preparation. LC could not be optimised because of the opposing influence of PLGA concentration on LE and LC. The outcome of the optimised parameter was calculated by the Minitab 18 software (Table 7). 

### 3.6. Verification of the Optimal Process Parameters

**Figure 5:**
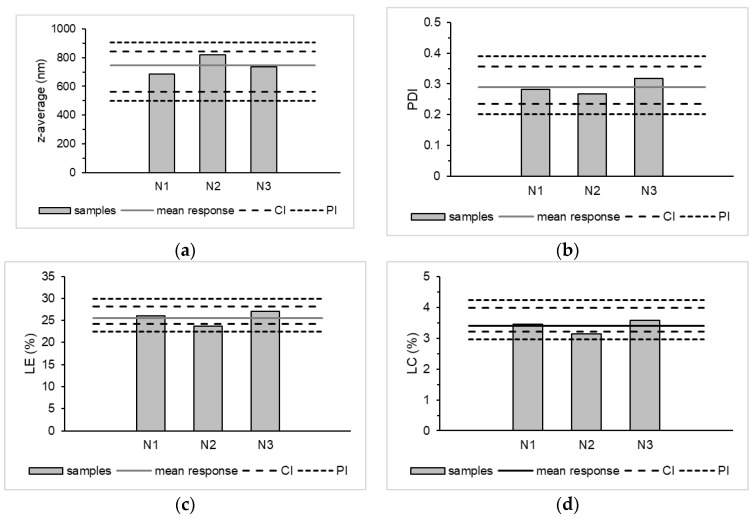
Verification of z-average (**a**), PDI (**b**), LE (**c**) and LC (**d**) of the product in triplicate and the 95 % confidence interval (CI) and 95 % prediction interval (PI).

To check whether the optimised parameters also provided the predicted result, the product was prepared in triplicate. The mean should be within the confidence interval (CI) and all individual values should be within the prediction interval (PI). The results can be seen in Figure 5, which confirmed the prognosis. The CCD was suitable for optimisation and adjustment of parameters and provided results within the 95% intervals.

The final product was characterised further by determination of the zeta-potential and visualisation by scanning electron microscopy. The zeta potential was −28.5 ± 0.6 mV. 

**Figure 6:**
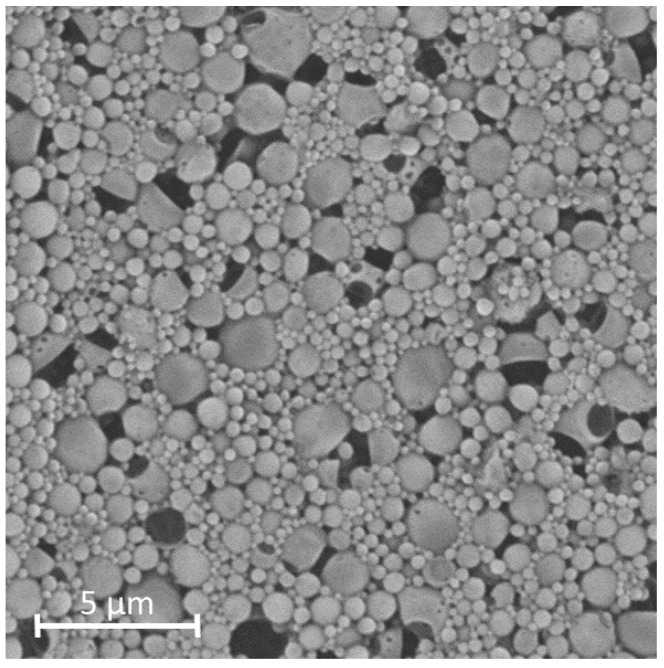
SEM-pictures of PLGA-NP produced with optimised parameters from the response surface design at 7000-fold magnification.

The SEM analysis (Figure 6) showed round particles with a certain size distribution, which confirmed the results from DLS measurements. 

This study did not address all the parameters to be considered in the development of a nanoparticulate PLGA formulation. Rather, an experimental example was chosen to illustrate the use of statistical experimental designs in parameter optimisation. Some factors (of composition and equipment) were assumed to be invariant and were thus not investigated further. For example, in a medicinal product, the choice of stabiliser and solvent would be considered critical to product quality, health, safety and environmental concerns, and would need to be clearly justified and controlled.

## 4. Conclusions

In this study, the preparation of OVA loaded PLGA-NP with a size of 700 nm for pulmonary delivery of antigens was utilised as model setup. They were successfully produced using several principles of Quality by Design. With the screening design, it was possible to gain general knowledge about which parameter influences which outcome, how significant this influence is, and in which direction it goes. With this knowledge, it was possible to regulate the outcomes within the limits of the design. If a target outcome is not covered by the screening design, it is still possible to estimate which parameter should be changed further. This makes a screening design a very suitable method for pretesting.

For further optimisation and precisely controllable outcomes, a response surface design was needed. With the CCD it was possible to detect interactions between the parameters and curvature. In the response surface plots, the outcomes for all different parameter combinations can be visualised. In the model setup, producing OVA-PLGA-NP with a double-emulsion solvent evaporation method, the parameters of the inner emulsion mainly influenced the drug loading (LE and LC) while parameters of the outer emulsion had an influence on size and size distribution. 

A pharmaceutical product should have well-defined quality attributes. As a measure of Quality by Design, a well understood and controllable manufacturing process is crucial, and critical process parameters should be known. The path shown here is a good way to achieve this goal with little effort.

## Figures and Tables

**Table 1:** Constant composition of double emulsion; concentrations varied.

Phase	Composition	Quantity
Internal aqueous phase (W1)	Ovalbumin in phosphate buffer, pH 7.4	1600 µL
Organic phase (O)	PLGA in ethyl acetate	4 mL
External aqueous phase (W2)	PVA in water	12 g
Stabiliser solution	PVA (1% (*w*/*w*)) in water	40 g

**Table 2:** Parameter settings of the definitive screening design.

Parameter	Low	Medium	High
c (OVA) [%]	2	3	4
c (PLGA) [%]	3	6	9
Stirring speed W/O	9500 rpm	-	20,500 rpm
Stirring time W/O [s]	30	60	90
c (PVA) [%]	2	3.5	5
Stirring speed W/O/W	8000 rpm	-	13,500 rpm
Stirring time W/O/W [s]	60	105	150

**Table 3:** Significance of parameters on responses and model summary.

	Outcome	z-Average	PDI	LE	LC
Parameter					
**OVA conc. (%)**		not sign.	not sign.	significant (*p* = 0.007)	significant (*p* = 0.000)
**PLGA conc. (%)**		significant (*p* = 0.0257)	not sign.	significant (*p* = 0.000)	significant (*p* = 0.000)
**w/o stirring speed (rpm)**		not sign.	not sign.	significant (*p* = 0.000)	significant (*p* = 0.002)
**w/o stirring time (s)**		not sign.	not sign.	not sign.	not sign.
**PVA conc. (%)**		significant (*p* = 0.00936)	not sign.	not sign.	not sign.
**w/o/w stirring speed (rpm)**		significant (*p* = 0.00893)	significant (*p* = 0.004)	not sign.	not sign.
**w/o/w stirring time (s)**		not sign.	not sign.	significant (*p* = 0.034)	not sign.
***p*** **-Value of model**		0.010	0.041	0.000	0.000
**R² (%)**		70.56	70.22	93.56	89.48

**Table 4:** Prognosis for optimised parameters of screening and results of the control samples.

Response	Fit	Result (n = 3) ± SD
z-Average (d.nm)	702	682.21 ± 11.94
PDI	0.1989	0.30 ± 0.03
LE (%)	19.88	24.25 ± 2.55
LC (%)	3.3299	4.07 ± 0.41

**Table 5:** Settings of central composite response surface design.

Parameter	Low Axial	Low	Medium	High	High Axial
c (PLGA) [%]	6.17	7	9	11	11.83
c (PVA) [%]	2.17	3	5	7	7.83

**Table 6:** Results of central composite response surface design.

Run	PLGA Conc. (%)	PVA Conc. (%)	z-Average (d.nm)	PDI	LE (%)	LC (%)
1	5	9	713.97	0.232	20.32	3.49
2	5	11.83	1204.67	0.353	27.17	3.54
3	3	11	1807.00	0.293	23.08	3.25
4	5	9	650.20	0.286	22.48	3.84
5	5	9	707.30	0.295	18.58	3.20
6	7	7	446.63	0.192	19.64	4.30
7	7.83	9	503.43	0.180	22.71	3.88
8	2.17	9	1810.33	0.311	19.53	3.36
9	5	9	606.73	0.255	23.21	3.96
10	7	11	565.50	0.257	25.20	3.54
11	5	6.17	506.23	0.242	18.20	4.51
12	3	7	873.43	0.351	18.90	4.14
13	5	9	695.43	0.301	22.61	3.86

**Table 7:** Prognosis for optimised parameters of response surface.

Response	Fit	95% CI	95% PI
z-Average (d.nm)	687.94	563.1; 812.7	496.0; 879.9
PDI	0.291	0.236; 0.347	0.201; 0.382
LE (%)	25.90	24.03; 27.77	22.24; 29.56
LC	3.57	3.22; 3.93	2.96; 4.19

## Data Availability

Data will be made available upon request.

## Supplementary Materials

The following supporting information can be downloaded at: https://www.mdpi.com/article/10.3390/pharmaceutics15020617/s1, Table S1: Results of definitive screening design.
