# Supplementary material for: Quality by Design as a Tool in the Optimisation of Nanoparticle Preparation—A Case Study of PLGA Nanoparticles
Source: Pharmaceutics. 2023 Feb 12;15(2):617. doi: 10.3390/pharmaceutics15020617 (PMC9966539; doi:10.3390/pharmaceutics15020617)
Supplement: Supplementary file 1 [file pharmaceutics-15-00617-s001.zip › pharmaceutics-2193059-supplementary.pdf]

Supplementary information

Table S1: Results of definitive screening design.

Run	Factors							Response			
	**OVA conc. (%)**	**PLGA conc. (%)**	**w/o stirring speed (rpm)**	**w/o stirring time (s)**	**PVA conc. (%)**	**w/o/w stirring speed (rpm)**	**w/o/w stirring time (s)**	**z-average (d.nm)**	**PDI**	**LE (%)**	**LC (%)**
1	2	3	9500	60	5	8000	150	558.83	0.366	11.52	2.98
2	4	9	9500	90	5	8000	105	1527.67	0.560	14.23	2.47
3	2	9	9500	30	5	13,500	60	594.13	0.374	15.05	1.32
4	2	9	20,500	90	2	8000	60	5565.00	1.000	22.68	1.98
5	4	9	20,500	60	2	13,500	60	2584.67	0.178	15.63	2.70
6	2	9	20,500	30	3.5	8000	150	1850.67	0.597	22.17	1.93
7	4	6	20,500	30	5	8000	60	1120.67	0.568	15.28	3.92
8	4	3	20,500	90	2	8000	150	2402.67	0.378	13.35	6.64
9	4	9	9500	30	2	8000	150	3304.33	0.271	16.72	2.89
10	2	3	20,500	90	5	13,500	60	443.90	0.280	13.13	3.38
11	2	6	9500	90	2	13,500	150	700.97	0.425	14.74	1.93
12	3	6	20,500	60	3.5	13,500	105	621.00	0.350	17.45	3.37
13	4	3	9500	90	3.5	13,500	60	596.90	0.401	8.10	4.14
14	3	9	20,500	90	5	13,500	150	689.13	0.360	23.95	3.09
15	4	3	9500	30	5	13,500	150	374.53	0.196	7.22	3.71
16	3	6	9500	60	3.5	8000	105	2129.67	0.583	11.55	2.26
17	2	3	20,500	30	2	13,500	105	510.73	0.331	15.24	3.90
18	3	3	9500	30	2	8000	60	1642.67	0.580	9.53	3.67
